# Effect of Citric Acid Concentration on the Transformation of Aragonite CaCO_3_ to Calcium Citrate Using Cockle Shells as a Green Calcium Source

**DOI:** 10.3390/ma18092003

**Published:** 2025-04-28

**Authors:** Pantita Chanwetprasat, Chaowared Seangarun, Somkiat Seesanong, Banjong Boonchom, Nongnuch Laohavisuti, Wimonmat Boonmee, Pesak Rungrojchaipon

**Affiliations:** 1 Material Science for Environmental Sustainability Research Unit, School of Science, King Mongkut’s Institute of Technology Ladkrabang, Bangkok 10520, Thailand; 66056054@kmitl.ac.th (P.C.); chaowared@gmail.com (C.S.); 2 Office of Administrative Interdisciplinary Program on Agricultural Technology, School of Agricultural Technology, King Mongkut’s Institute of Technology Ladkrabang, Bangkok 10520, Thailand; somkiat.se@kmitl.ac.th (S.S.); 3 Municipal Waste and Wastewater Management Learning Center, School of Science, King Mongkut’s Institute of Technology Ladkrabang, Bangkok 10520, Thailand; 4 Department of Chemistry, School of Science, King Mongkut’s Institute of Technology Ladkrabang, Bangkok 10520, Thailand; pisak.ru@kmitl.ac.th; 5 Department of Biology, School of Science, King Mongkut’s Institute of Technology Ladkrabang, Bangkok 10520, Thailand; wimonmat.bo@kmitl.ac.th

**Keywords:** calcium citrate, Ca_3_(C_6_H_5_O_7_)_2_·4H_2_O, CaCO_3_, cockle shell waste, citric acid, productivity

## Abstract

Aragonite calcium carbonate (CaCO_3_), derived from cockle shell waste, was successfully used as a renewable calcium source to synthesize calcium citrate (CCT) using citric acid (C_6_H_8_O_7_). The three CCT products (CCT-2, CCT-3, and CCT-4) were prepared using three different acid concentrations: 2, 3, and 4 M. The physicochemical characteristics of the newly synthesized CCT were investigated. Fourier-transform infrared (FTIR) spectra revealed the vibrational modes of the citrate anionic group (C_6_H_5_O_7_^3−^), which preliminarily confirmed the characteristics of CCT. However, X-ray diffraction (XRD) revealed that the concentration of citric acid altered the structural property and the chemical formula of the synthesized CCT. Employing 2 M citric acid, a pure tetra-hydrated phase (Ca_3_(C_6_H_5_O_7_)_2_·4H_2_O, earlandite mineral) was obtained. However, a mixture of hydrated (Ca_3_(C_6_H_5_O_7_)_2_·4H_2_O) and anhydrous (Ca_3_(C_6_H_5_O_7_)_2_) phases was precipitated when 3 and 4 M citric acid was used in the preparation process. The lower mass loss observed in the thermogravimetric analysis (TGA) of CCT-3 and CCT-4 compared to that of CCT-2 further confirmed that CCT-3 and CCT-4 were composed of hydrated and anhydrous CCTs. The synthesized CCT decomposed in four major processes: the first dehydration, the second dehydration, CaCO_3_ formation, and decarbonization, generating calcium oxide (CaO) as the final product. X-ray fluorescence (XRF) results showed that the CCT mainly consisted of CaO with a quantity of >98%. The scanning electron microscopic (SEM) image revealed the irregular plate-like CCT crystallites. The concentration of citric acid is a key factor that influences the productive parameters of CCT, including production yield, reaction time, and solubility. 2 M citric acid provided the optimal balance between productivity and cost-effectiveness, with the highest yield and soluble fraction and the lowest reaction time. The results suggest that the preparation of CCT from cockle shell waste can potentially replace the use of commercial calcite from mining, which is a limited and non-renewable resource.

## 1. Introduction

Calcium citrate (CCT) is a critical organic calcium salt that can be applied in many industries, especially medicine [1] and food supplements [2]. CCT is often used as a calcium supplement because it is more bioavailable than calcium carbonate (CaCO_3_) [3], one of the most common forms of calcium. CCT has been used in biomaterial fields such as rhBMP-2, which has shown a beneficial effect on osteoinduction and osteogenesis [4]. In nature, calcium citrate tetrahydrate (Ca_3_(C_6_H_5_O_7_)_2_·4H_2_O) has been observed as an earlandite mineral in unconsolidated ocean floor sediment [5]. CCT compounds are typically synthesized through a chemical process using CaCO_3_ [6], calcium chloride (CaCl_2_) [7], or calcium nitrate tetrahydrate (Ca(NO_3_)_2_·4H_2_O) [8] as raw materials. It is also prepared by a microbial biomineralization process using fungi [9]. Among these raw materials, CaCO_3_ is a common mineral form of calcium compound with the lowest value but the highest availability. CaCO_3_ is widely used as a raw material for preparing value-added calcium materials, such as calcium lactate pentahydrate (Ca(CH_3_CHOHCOO)_2_·5H_2_O) [10].

CaCO_3_ has three anhydrous crystalline polymorphs: hexagonal vaterite, orthorhombic aragonite, and rhombohedral calcite [11]. Calcite is the most abundant polymorph and can be found in numerous sources, such as limestone, eggshells, and some marine shells, such as scallops, oysters, and mussel shells [12,13,14]. In contrast, the aragonite polymorph is mainly observed in a few organisms, such as cuttlebone, cockle shells, clam shells, and mussel shells [15]. Moreover, the crystal structure of CaCO_3_ has received much attention because of its industrial and scientific importance [16,17]. Among the three polymorphs, calcite is the most stable polymorph of CaCO_3_ under ambient conditions, whereas aragonite is metastable and vaterite is the most unstable polymorph [18,19,20]. In shellfish, both aragonite and calcite are found, depending on the species [21].

Due to its unique physicochemical and structural properties, aragonite CaCO_3_ is of interest for utilization as a raw material to prepare value-added calcium compounds [10]. Suwannasingha et al. [15] studied the preparation of calcium oxide (CaO) from various calcium sources and reported that aragonite CaCO_3_ from cuttlebone was completely converted to CaO at 700 dC, while calcite CaCO_3_ from oyster shells required a temperature of 900 °C to become CaO completely. This finding suggests that the utilization of aragonite CaCO_3_ as a calcium source is an effective pathway to achieve value-added calcium compounds, such as CaO, with lower energy consumption. CaO is the most effective alkaline metal oxide catalyst for biodiesel production from different oil resources [22]. The reaction time for preparing hydrated calcium lactate (Ca(CH_3_CHOHCOO)_2_·5H_2_O) from cockle shells, which consist of only aragonite CaCO_3_, is significantly lower than that from mussel shells and oyster shells, which mainly consist of calcite CaCO_3_ [10]. Moreover, the production yield of value-added calcium compounds from cockle shells is higher than that obtained from other shells due to the high purity of the calcium sources. Therefore, this research aims to synthesize calcium citrate (CCT) through a mild, simple, and rapid process using citric acid (C_6_H_8_O_7_) and aragonite CaCO_3_, derived from cockle shell waste, as the sustainable calcium source. The effect of C_6_H_8_O_7_ concentration on the physicochemical properties of the synthesized CCT was also investigated. The findings observed in this research highlighted the potential of the green synthesis technique with optimization for the productivity of CCT, resulting in a reduction in the production cost for industrial-scale applications. Moreover, waste recycling reduces the greenhouse effect during the production process, which is consistent with the world’s carbon neutrality trend and sustainable development goals (SDGs) [23,24].

## 2. Materials and Methods

### 2.1. Raw Material Preparation

Cockleshell wastes were used as a renewable calcium source instead of commercial CaCO_3_ from mining to reduce the use of natural resources and environmental problems from shell waste [10,15]. Cockle shells used in this research were obtained from a coastal landfill in Chonburi Province, Thailand. The collected shells were washed, dried, ground, and sieved through a 100-mesh sieve to obtain aragonite CaCO_3_ powder [25]. Three different citric acid (C_6_H_8_O_7_) concentrations, 2, 3, and 4 M, were prepared by dissolving the desired amount of commercial-grade citric acid powder (99% Fisher Chemical™,UK) in DI water.

### 2.2. Calcium Citrate (CCT) Preparation

The obtained aragonite CaCO_3_ powders were converted to calcium citrate (CCT) (Ca_3_(C_6_H_5_O_7_)_2_) in the presence of C_6_H_8_O_7,_ according to Equation (1) [2]. The effect of C_6_H_8_O_7_ concentration (2, 3, and 4 M) on the physicochemical characteristics of the prepared CCT was investigated to optimize the parameters of the synthesis.

3CaCO_3_(s) + 2C_6_H_8_O_7_(aq) → Ca_3_(C_6_H_5_O_7_)_2_(s) + 3H_2_O(l) + 3CO_2_(g) ↑ (1)

The preparation process of CCT was carried out by weighing 10 g of powdered CaCO_3_ and placing it in a glass beaker. Then, 33.30 mL of 2 M citric acid concentration (22.22 mL of 3 M or 16.67 mL of 4 M) was slowly added to each beaker containing powdered CaCO_3_. The mixture was stirred (300 rpm) until no carbon dioxide (CO_2_) evolved, indicating a complete synthesis reaction. After that, the mixture was exposed to atmospheric conditions until it was completely dried, resulting in the formation of white CCT powders. The obtained products were labeled as CCT-2, CCT-3, and CCT-4 for the samples synthesized using 2, 3, and 4 M citric acid solution, respectively. Triplicate experiments were performed for each citric acid solution. Three essential parameters were measured and calculated: reaction time (time for complete synthesis reaction), soluble fraction (the percentage calculated from the mass ratio between the residual CCT mass and the initial mass of CCT used in the test), and percentage yield (the amount ratio between the obtained product (CCT) and utilized precursor (cockle-shell-derived CaCO_3_).

### 2.3. Material Characterizations

The functional group in the synthesized CCT sample was analyzed using a Fourier transform infrared (FTIR) spectrophotometer (Spectrum GX, PerkinElmer, Waltham, MA, USA). The spectral profile was recorded in the wavenumber range of 400–4000 cm^−1^ at a resolution of 1 cm^−1^. The crystal structure and phase purity of the samples were characterized by X-ray diffraction (Bruker AXS, Billerica, MA, USA) using Cu-K_α_ radiation (λ = 0.15406 nm). The XRD pattern of the sample was analyzed at 2*θ* angles from 5–60° with an increment of 0.01° at a scan speed of 1 s/step [26]. The obtained diffraction was compared with the Joint Committee on Powder Diffraction Standards (JCPDS) database [27] to clarify the crystalline characteristics and phase purity. The thermal decomposition of the sample was analyzed using thermogravimetric analysis (TG/DTA Pyris Diamond, PerkinElmer, Waltham, MA, USA). The sample (~10 mg) was placed in a calcined alumina crucible without a lid and thermally treated at a constant heating rate of 10 °C/min from room temperature to 900 °C at a constant N_2_ gas flow rate of 100 mL/min [28]. The thermally induced mass loss and derivative thermogravimetric characteristics were then interpreted and discussed. The surface morphology and elemental composition of the sample were further characterized by scanning electron microscopy (SEM, VP1450, LEO, North Billerica, MA, USA). [7] and X-ray fluorescence (XRF, SRS 3400, Bruker, Billerica, MA, USA) [29], respectively. Prior to SEM operation, the sample was coated with gold to increase its conductivity and reduce the charging effect [30]. The solubility of the products was investigated as follows: A typical process involved dissolving 10 g of the CCT sample in 100 mL of DI water, followed by continuous stirring at 100 rpm at room temperature for 1 h. The insoluble fraction(solid) was separated by filtration using a suction pump and dried in an oven (100 °C for 1 h) to determine the weight of the dried solid, which was used to estimate the % solubility. Triplicate experiments were performed for each sample. 

## 3. Results and Discussion

### 3.1. Functional Groups of CCT

**Figure 1:**
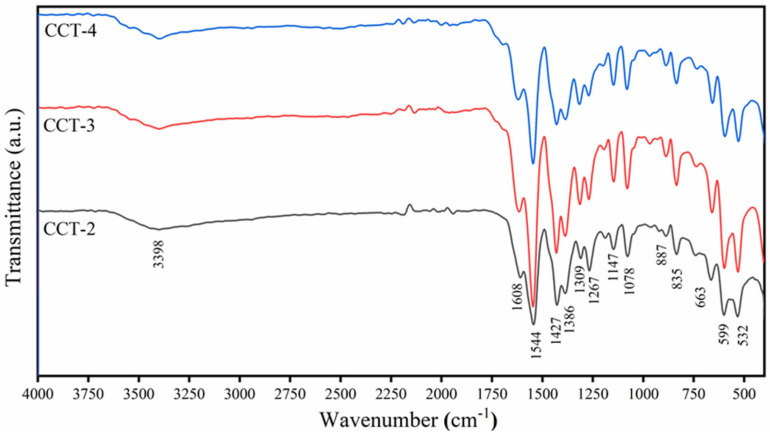
Infrared absorption spectra of calcium citrate derived from aragonite CaCO_3_ using 2 (CCT2), 3 (CCT3), and 4 (CCT4) M citric acid.

Due to the transmission/absorption of infrared radiation, the Fourier transform infrared (FTIR) technique was utilized to identify the functional groups that existed in the material [31]. Figure 1 shows the FTIR spectra of the CCT products synthesized in this study. Similar spectral patterns across different CCT-2, CCT-3, and CCT-4 samples indicate the presence of the same functional groups in each CCT sample. The FTIR spectra revealed that the functional groups of CCT prepared from the cockle-shell-derived aragonite CaCO_3_ were consistent with those of CCT products prepared from both CaCO_3_ [9] and CaCl_2_ [7] precursors, preliminarily indicating the successful preparation of CCT in this work.

The vibrational modes of the functional groups are described in detail below. The absorption broad band located at 3398 cm^−1^ is attributed to O–H stretching, confirming the presence of H_2_O in the CCT crystal structure and/or the moisture absorbed on the sample surface. Two intense absorption peaks observed between 1700 and 1350 cm^−1^ were assigned to the vibrational characteristics of the carboxyl group (–COOH) in all samples. The carboxyl group in CCT can coordinate with Ca^2+^ ions [32]. The characteristic peaks observed at 1608 and 1544 cm^−1^ indicate the presence of the antisymmetric stretching vibration of the carboxylate group (–COO^–^), while the characteristic peaks observed at 1427 and 1386 cm^−1^ are the symmetric stretching vibrations of–COO^–^ [7]. The absorption bands at 1309 and 1267 cm^−1^ corresponded to the symmetric C–H bending vibration of the citrate ions (C_6_H_5_O_7_^3−^ or C_3_H_5_O(COO)_3_^3−^). The characteristic peak at 1078 cm^−1^ is assigned to the C–O stretching vibration of C_3_H_5_O(COO)_3_^3−^. The absorption peaks observed at 887 and 835 cm^−1^ are related to the C–C stretching of C–COO^–^. The peaks at 599 and 532 cm^−1^ corresponded to the out-of-plane O=C–O bending vibration. These preliminary spectroscopic results suggested that the synthesized product was CCT. However, the exact chemical formula and crystal structure of the product (Ca_3_(C_6_H_5_O_7_)_2_ or Ca_3_(C_6_H_5_O_7_)_2_·4H_2_O) could not be determined using this characterization technique, and other direct techniques are required to solve this issue.

### 3.2. Crystal Structure of CCT

**Figure 2:**
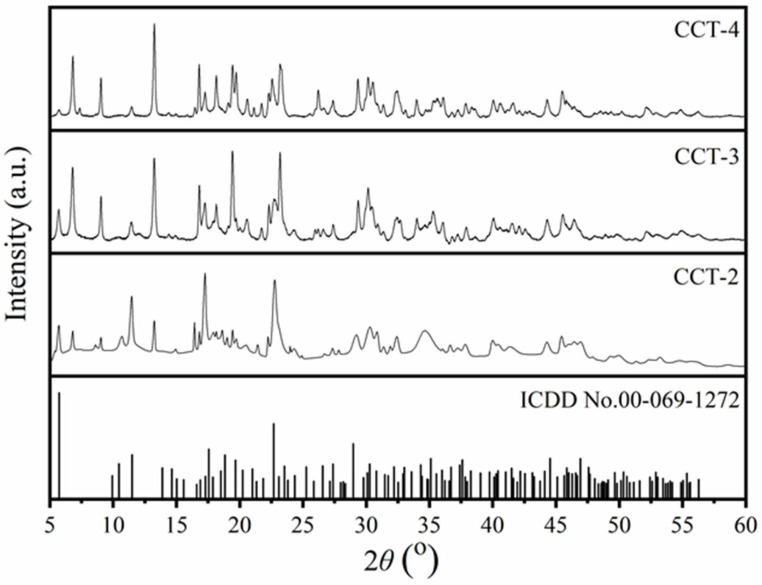
X-ray diffraction patterns of calcium citrate derived from aragonite CaCO_3_ using 2 (CCT2), 3 (CCT3), and 4 (CCT4) M citric acid compared with the standard ICDD #00-069-1272 of Ca_3_(C_6_H_5_O_7_)_2_·4H_2_O.

X-ray diffraction (XRD) is an effective technique for determining the crystal structure and phase purity of a material [33]. Figure 2 shows the XRD patterns of CCT synthesized from aragonite CaCO_3_ using 2 (CCT2), 3 (CCT3), and 4 (CCT4) M citric acid. The diffraction patterns of CCT-2, CCT-3, and CCT-4 corresponded well to the standard file of the earlandite mineral [9] or tri-calcium di-citrate tetrahydrate (Ca_3_(C_6_H_5_O_7_)_2_·4H_2_O) according to ICCD no. 00-069-1272, confirming the successful synthesis of hydrated CCT (Ca_3_(C_6_H_5_O_7_)_2_·4H_2_O) using aragonite CaCO_3_. The triclinic crystal structure of Ca_3_(C_6_H_5_O_7_)_2_·4H_2_O was reported by Herdtweck et al. [8] with a space group of *P1*. The lattice parameters are *a* = 5.9466 Å, *b* = 10.2247 Å, and *c* = 16.6496 Å. The lattice angles are *α* = 72.213°, *β* = 79.718°, and *γ* = 89.791°. The volume of the unit cell and the number of molecules or formula units (*Z*) in the unit cell were 947.06 Å^3^ and 2, respectively.

It was also observed that the diffraction peaks of 2*θ* at 5° and 11° for CCT-3 and CCT-4 samples are relatively lower than that of the CCT-2 sample. These diffraction peaks (2*θ* at 5° and 11°) corresponded to the (001) and (002) planes of the hydrated form (Ca_3_(C_6_H_5_O_7_)_2_·4H_2_O). However, higher intensities of the diffraction peaks at 6° and 9° were observed for CCT-3 and CCT-4. Therefore, it could be interpreted that the concentration of citric acid significantly affected the crystal structure of the synthesized CCT. The excess citric acid used in CCT preparation could disturb the formation of CCT, leading to the generation of a side reaction, as shown in Equation (2):

Ca_3_(C_6_H_5_O_7_)_2_ (CCT) + C_6_H_8_O_7_ → 3CaH(C_6_H_5_O_7_) (2)

This side reaction causes the deformation of CCT and the formation of calcium hydrogen citrate (CaH(C_6_H_5_O_7_)) [2]. Liu et al. [34] also reported that the diffraction peaks at 6° and 9° were identified as the diffraction peaks of anhydrous CCT (Ca_3_(C_6_H_5_O_7_)_2_). Therefore, it could be mentioned that the synthesized CCT product mainly consisted of the tetra-hydrated form (Ca_3_(C_6_H_5_O_7_)_2_·4H_2_O) with the partial anhydrous form (Ca_3_(C_6_H_5_O_7_)_2_). The lowest diffraction intensity at 6° and 9° for CCT-2 indicates the complete precipitation of Ca_3_(C_6_H_5_O_7_)_2_·4H_2_O, compared to both CCT-3 and CCT-4. The imperfections in the crystal structures of CCT-3 and CCT-4 can be explained by the lower amount of water and the high viscosity of concentrated citric acid in the reaction. In addition, hydrated CCT (Ca_3_(C_6_H_5_O_7_)_2_·4H_2_O) is more stable than anhydrous CCT, indicating that CCT-2 is consistent with market demand [2].

### 3.3. Thermal Decomposition of CCT

**Figure 3:**
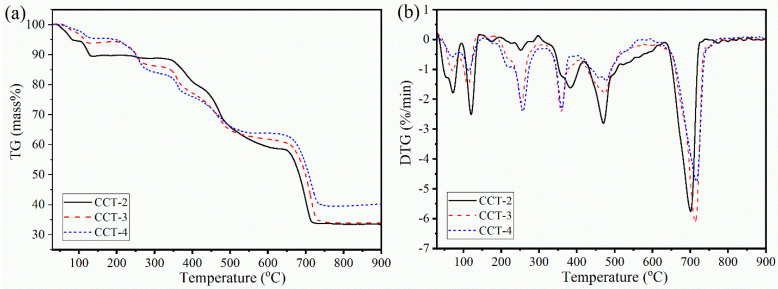
Thermal decomposition behaviors (TG (**a**) and DTG (**b**) curves) of CCT-2, CCT-3, and CCT-4 derived from aragonite CaCO_3_ using 2, 3, and 4 M citric acid.

A thermal analysis experiment was performed to investigate the thermal decomposition behavior [35] of the synthesized CCT, and the resulting thermogravimetric (TG) and derivative thermogravimetric (DTG) profiles were interpreted. Figure 3 shows the thermal decomposition process of the CCT samples measured in the temperature range of 30–900 °C.

Both the TG (Figure 3a) and DTG (Figure 3b) curves of CCT-2 are significantly different from those of CCT-3 and CCT-4. These different thermal decompositions corresponded to the difference in the water content in the crystal structure between CCT-2 and CCT-3 or CCT-4, which is consistent with the difference in the XRD patterns (Figure 2). However, the major decomposition step for all the studied samples revealed similar profiles, as demonstrated by the DTG curves. The thermal decomposition of each sample was divided into four main steps, corresponding to the four different TG mass losses, through the following equations:

First dehydration (30–150 °C):

Ca_3_(C_6_H_5_O_7_)_2_·4H_2_O(s) → Ca_3_(C_6_H_5_O_7_)_2_·2H_2_O(s) + 2H_2_O(g) ↑ (3)

Second dehydration (150–330 °C):

Ca_3_(C_6_H_5_O_7_)_2_·2H_2_O(s) → Ca_3_(C_6_H_5_O_7_)_2_(s) + 2H_2_O(g) ↑ (4)

Decomposition to CaCO_3_ (330–600 °C):

Ca_3_(C_6_H_5_O_7_)_2_(s) → 3CaCO_3_(s) + C_9_H_10_O_5_(g) ↑ (5)

Decarbonization (600–730 °C):

3CaCO_3_(s) → 3CaO(s) + 3CO_2_(g) ↑ (6)

The mass loss in the first dehydration step (Equation (3)), which appeared in the temperature range from 30 to 150 °C, corresponded to the removal of surface-adsorbed water molecules and a part of the crystal water molecules (water of crystallization) [7]. Two-mole equivalents of water (2H_2_O) were removed, and the dihydrated product (Ca_3_(C_6_H_5_O_7_)_2_·2H_2_O) was obtained. The second mass loss (Equation (4), second dehydration step), which occurred at temperatures between 150 and 330 °C, is attributed to the elimination of the remaining constituent water (2H_2_O). In these first and second dehydration processes, both the adsorbed water on the surface and constituent water (4H_2_O) were completely removed, and the dehydrated CCT form (anhydrous Ca_3_(C_6_H_5_O_7_)_2_) was obtained [9]. The mass loss of the first and second dehydration processes was evaluated from the TG curve [9,34,35] and was found to be ~10% for the CCT-2 sample, which is slightly lower than the theoretical value of 12.63%. The lower mass loss was related to the presence of absorbed water. However, the mass loss in the first and second dehydrations of CCT-3 and CCT-4 (~4%) was significantly lower than that of CCT-2. The results were consistent with the XRD results, corresponding to the formation of anhydrous Ca_3_(C_6_H_5_O_7_)_2_, resulting in the presence of mixed phases between Ca_3_(C_6_H_5_O_7_)_2_·4H_2_O and Ca_3_(C_6_H_5_O_7_)_2_. The mixture of these hydrated and anhydrous crystal forms led to a reduction in the mass loss of both CCT-3 and CCT-4 compared to that of CCT-2.

In addition, the decomposition behavior of the synthesized Ca_3_(C_6_H_5_O_7_)_2_·4H_2_O also indicated the environmental difference of the constituent water (4H_2_O) in the crystal structure. According to the dehydration process, 2H_2_O was first thermally eliminated from tetra-hydrated CCT (Ca_3_(C_6_H_5_O_7_)_2_·4H_2_O), followed by the removal of the remaining two molecules of H_2_O from Ca_3_(C_6_H_5_O_7_)_2_·2H_2_O, forming anhydrous Ca_3_(C_6_H_5_O_7_)_2_. Therefore, the exact chemical formula of hydrated calcium citrate can be rewritten as [Ca_3_(C_6_H_5_O_7_)_2_(H_2_O)_2_]·2H_2_O, which is consistent with the findings in the literature [7,8]. This could also be interpreted as the inner water, or (H_2_O)_2_ in the [Ca_3_(C_6_H_5_O_7_)_2_(H_2_O)_2_]·2H_2_O, chemically interacting with Ca_3_(C_6_H_5_O_7_)_2_ with a stronger interaction than the outer water, or 2H_2_O. In this case, the outer water was first thermally removed, followed by the removal of the inner water, which is consistent with the TG/DTG profile of CCT, especially CCT-2.

A TG mass loss of ~34% in the third step (decomposition to CaCO_3_) with three DTG peaks was observed at the temperature from 330 to 600 °C. This thermal step was assigned to the thermal decomposition of anhydrous Ca_3_(C_6_H_5_O_7_)_2_, associated with the formation of CaCO_3_ (Equation (5)) [7,9]. In this thermal decomposition step, syringic acid (C_9_H_10_O_5_) [36,37] was eliminated as CO, CO_2_, and H_2_O [7,8]. The final thermal decomposition step with a mass loss of ~25% appeared in the temperature range from 630 to 730 °C. This step is related to the decomposition of CaCO_3_ with the elimination of CO_2_ (decarbonization), as expressed in Equation (6), resulting in the formation of CaO as the final decomposition product. The TG mass loss in the third and final steps was also close to the theoretical values of 34.74% and 23.16%, respectively. The thermal decomposition characteristics of all Ca_3_(C_6_H_5_O_7_)_2_·4H_2_O samples prepared from cockle-shell-derived aragonite CaCO_3_ in this work were similar to those of Ca_3_(C_6_H_5_O_7_)_2_·4H_2_O prepared from oyster-shell-derived calcite CaCO_3_ [15]. However, the complete thermal temperature of the sample obtained in this work was found to be ~730 °C, which is slightly lower than that of the sample prepared from calcite (900 °C) [15].

### 3.4. Purities of CCT Determined by XRF

X-ray fluorescence (XRF), a non-destructive testing technique, was used to evaluate the elemental composition of the solid-state sample (CCT) synthesized in this work [38]. A secondary fluorescent emission profile was observed owing to the excitation of atomic inner-shell electrons by the primary X-ray beam [39]. Table 1 lists the chemical compositions of the synthesized CCT samples determined using the XRF technique. The results showed that all synthesized CCT-2, CCT-3, and CCT-4 samples mainly consisted of CaO with the same wt% value (main composition, 98.2 wt%). However, other metal oxides were also observed at approximate amounts in all samples (minor compositions, 1.8 wt%).

The experimental results observed in this work highlighted that cockle shell waste is an alternative renewable calcium source for preparing high-purity CCT without toxic elements, such as cadmium (Cd), arsenic (As), mercury (Hg), and lead (Pb). High-quality CCT products prepared from cockle shells (aragonite CaCO_3_) have attracted increasing attention in the food industry as both calcium supplements and food additives [2]. The synthesized CCT product could also be applied for bone reparation [25] due to its faster absorbability than calcium phosphate [40] and hydroxyapatite [41].

### 3.5. Morphologies of CCT

**Figure 4:**
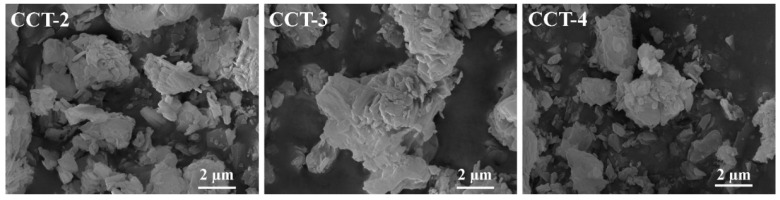
SEM images at a magnification of 20k× of CCT particles derived from aragonite CaCO_3_ using 2, 3, and 4 M citric acid.

The scanning electron microscope (SEM), a type of electron microscope, generates the sample surface images using a focused electron beam, which reveals the morphological characteristics of the imaged sample [42]. Figure 4 illustrates the morphologies of the CCT samples synthesized from aragonite CaCO_3_ in the presence of different citric acid concentrations. Morphological analysis revealed that the CCT synthesized from aragonite crystals presented irregular plate-like crystallites that aggregated into enlarged irregular particles. The dimensions of the particles are in the range of 0.3–2 µm. The SEM images of CCT-2, CCT-3, and CCT-4, synthesized from the aragonite CaCO_3_ precursor, presented morphological characteristics similar to those of the earlandite (Ca_3_(C_6_H_5_O_7_)_2_·4H_2_O) synthesized from the calcite CaCO_3_ precursor [9], indicating the successful preparation of Ca_3_(C_6_H_5_O_7_)_2_·4H_2_O.

### 3.6. Preparation Results

Equation (1) represents the preparation of CCT (Ca_3_(C_6_H_5_O_7_)_2_), which was obtained through reactive crystallization [2]. During the reaction, as shown in the equation, 3Ca^2+^ and 2C_6_H_5_O_7_^3−^ ions were solvated by water with the elimination of CO_2_. After that, the solvent (water) could evaporate from the reaction, causing the crystallization process in a supersaturation situation. The saturated point induced the formation of a 3Ca^2+^-2C_6_H_5_O_7_^3−^ nucleus, followed by the Ca_3_(C_6_H_5_O_7_)_2_ crystal growth process. However, the hydrated form of the product was also fabricated due to the presence of an aqueous-based citric acid solution, resulting in the formation of Ca_3_(C_6_H_5_O_7_)_2_·4H_2_O or [Ca_3_(C_6_H_5_O_7_)_2_(H_2_O)_2_]·2H_2_O. Consequently, Ca_3_(C_6_H_5_O_7_)_2_·4H_2_O was obtained in this work using a cockle-shell-derived aragonite CaCO_3_ precursor, and the effect of citric acid concentration (2, 3, and 4 M) on the physicochemical properties of the products was investigated using FTIR, XRD, TG/DTG, and SEM.

In addition, after applying the same preparation conditions, the parameters investigated, including the production yield, reaction time, and soluble fraction of all prepared CCT samples, were obtained, and the results are reported in Table 2.

It was found that the reaction time increased with increasing citric acid concentration due to the higher amount of citrate ions (C_6_H_5_O_7_^3−^), which increased the viscosity of the synthesis reaction. However, at high citric acid concentrations, the reaction formed CCT precipitation with a rigid structure, causing a low soluble fraction percentage. The production yields were calculated to be 98.57%, 93.14%, and 88.63% when 2, 3, and 4 M citric acid solutions were used in the production reaction, respectively. The lowest yield obtained from the highest citric acid concentration was consistent with the longer reaction time (85 min). The high viscosity and density of citric acid [43] compared to water inhibited the production progress of the CCT product, resulting in a decreased production yield.

It was also observed that an increase in citric acid concentration caused the formation of a higher proportion of anhydrous CCT (Ca_3_(C_6_H_5_O_7_)_2_) in the mixed precipitate products (Ca_3_(C_6_H_5_O_7_)_2_·4H_2_O + Ca_3_(C_6_H_5_O_7_)_2_). The increased proportion of anhydrous Ca_3_(C_6_H_5_O_7_)_2_ was verified and discussed in the XRD and TG/DTG results. The results suggest that the concentration of citric acid is the key factor [44] that significantly impacts the CCT crystal forms (hydrated or anhydrous product) and their physicochemical properties, as well as the production yield, reaction time, and soluble fraction of CCT. From the results obtained, it can be concluded that 2 M citric acid is the optimum condition for preparing hydrated calcium citrate (Ca_3_(C_6_H_5_O_7_)_2_·4H_2_O) with the highest yield percentage, lowest reaction time, and highest soluble fraction. The CCT products in this work were obtained with a short time consumption (<85 min), high yield, and high purity of the product, and the differences in some properties from calcium citrate (earlandite) synthesis using microorganisms [7,8,9]. This makes the product obtained by this method low-cost compared with those obtained using other synthesis methods [7,8,9]. Furthermore, the obtained hydrated CCT is considered to be the CCT form that meets the market demand for application as a calcium supplement and food additive [2], and for bone reparation [25].

## 4. Conclusions

With the addition of citric acid (C_6_H_8_O_7_), calcium citrate (CCT, Ca_3_(C_6_H_5_O_7_)_2_) was successfully synthesized using aragonite CaCO_3_, derived from cockle shell waste as a green calcium source. Three different C_6_H_8_O_7_ concentrations (2, 3, and 4 M) were used in the synthesis, and CCT-2, CCT-3, and CCT-4 products were obtained, respectively. The vibrational spectroscopy of all CCT, revealed by FTIR spectra, shows the same functional groups corresponding to the citrate anion. However, the XRD patterns and thermal decomposition (TG/DTG) profiles revealed differences in the number of constituent water molecules in the Ca_3_(C_6_H_5_O_7_)_2_ crystal structure. Using 2 M citric acid, Ca_3_(C_6_H_5_O_7_)_2_·4H_2_O or [Ca_3_(C_6_H_5_O_7_)_2_(H_2_O)_2_]·2H_2_O was obtained for CCT-2, whereas an anhydrous Ca_3_(C_6_H_5_O_7_)_2_ phase was further observed by using 3 and 4 M citric acid, thereby obtaining the mixture between [Ca_3_(C_6_H_5_O_7_)_2_(H_2_O)_2_]·2H_2_O and Ca_3_(C_6_H_5_O_7_)_2_. The mass loss of the first and second dehydrations of CCT-3 and CCT-4 were significantly lower than those of CCT-2. These findings are consistent with the XRD results. However, the constituent water did not alter the morphological characteristics of the as-prepared CCT products. The XRF results revealed the high purity of the CCT products, which could be further considered as food supplements and bone reparations. Based on the production yield, reaction time, and soluble fraction percentage, the optimum concentration of citric acid was determined to be 2 M. All results highlighted that aragonite CaCO_3_ derived from cockle shell waste could be effectively utilized as a renewable calcium source, instead of limited and non-renewable calcium ores, to synthesize CCT product. This has led to the development of green and sustainable techniques to reduce natural resource use and shell waste, which have environmental implications.

## Figures and Tables

**Table 1:** Elemental compositions of CCT-2, CCT-3, and CCT-4 were analyzed by X-ray fluorescence (XRF) technique.

Elemental Compositions		Elemental Contents/wt%		
		CCT-2	CCT-3	CCT-4
Major composition				
Calcium oxide	CaO	98.1778	98.1805	98.1857
Minor compositions				
Sodium oxide	Na_2_O	0.8520	0.8200	0.8410
Magnesium oxide	MgO	0.0462	0.0414	0.0470
Aluminum oxide	Al_2_O_3_	0.0323	0.0331	0.0431
Silicon dioxide	SiO_2_	0.0854	0.0903	0.0925
Phosphorus pentoxide	P_2_O_5_	0.0193	0.0187	0.0209
Sulfur trioxide	SO_3_	0.1370	0.1330	0.1430
Chlorine	Cl	0.0212	0.0192	0.0211
Potassium oxide	K_2_O	0.0123	0.0097	0.0126
Cobalt oxide	Co_2_O_3_	-	0.0211	-
Zinc oxide	ZnO	-	0.0146	-
Strontium oxide	SrO	0.4350	0.4450	0.455
Ferrous oxide	Fe_2_O_3_	0.1470	0.1450	0.1460
Summations		~100	~100	~100

**Table 2:** The production parameters of CCT-2, CCT-3, and CCT-4 from aragonite CaCO_3_.

Samples	Reaction Time (min)	Production Yields (%)	Soluble Fractions (%)
CCT-2	54	98.57 ± 1.46	92.51 ± 1.24
CCT-3	67	93.14 ± 1.37	88.72 ± 1.33
CCT-4	85	88.63 ± 1.42	84.17 ± 1.29

## Data Availability

Data are contained within the article.
